# The Role of Urate in Cardiovascular Risk in Adolescents and Young Adults With Hypertension, Assessed by Pulse Wave Velocity

**DOI:** 10.3389/fcvm.2022.867428

**Published:** 2022-04-15

**Authors:** Mirjam Močnik, Sonja Golob Jančič, Martina Filipič, Nataša Marčun Varda

**Affiliations:** Division of Paediatrics, University Clinical Centre Maribor, Maribor, Slovenia

**Keywords:** urate, adolescents and young adults, hypertension, obesity, cardiovascular risk, pulse wave velocity

## Abstract

**Background:**

Urate is increasingly recognized as a cardiovascular risk factor. It has been associated with hypertension, metabolic syndrome, obesity, chronic kidney disease and diabetes. Its prognostic role is less clear. The aim of our study was to evaluate the association between serum urate and pulse wave velocity, a measure of arterial stiffness in hypertensive adolescents and young adults.

**Methods:**

269 adolescents and young adults with hypertension were included in the study. From all, anthropometric, blood pressure, pulse wave velocity and serum urate measurements were made. Variables were compared between sex, participants with or without obesity and with or without elevated urate.

**Results:**

In multiple regression analysis for urate as dependent variable gender and diastolic pressure were found to be statistically significant. The difference between urate levels were found between boys and girls (*p* < 0.001), obese and non-obese (*p* < 0.001); however, pulse wave velocity did not differ between hyper- and eu-uricemic group (*p* = 0.162).

**Conclusion:**

Associations between urate, gender, diastolic blood pressure and obesity were confirmed, however, no significant associations between pulse wave velocity and urate were detected.

## Introduction

Urate is a breakdown product of human purine metabolism and has been widely associated with metabolic syndrome, hypertension, cardiovascular diseases, and chronic kidney disease in both adult and pediatric populations (1, 2).

The serum urate levels depend on dietary intake of purines, the degradation of endogenous purines and the renal and intestinal excretion of urate (3), therefore the question was raised in the past if associations are merely a consequence of greater purine intake or urate is truly an independent factor for cardiovascular risk.

Some further studies showed urate's independent role in development of hypertension, stroke and heart failure, as well as emerging role in the pathogenesis of kidney disease, metabolic syndrome and diabetes (4). Recent findings support an association between elevated urate level and the risk of arterial hypertension from epidemiological, clinical and experimental point of view (5). A significant, but modest association between urate and cardiovascular disease or mortality was demonstrated. An increased risk for chronic heart disease incidence and mortality were more pronounced in hyperuricemic women (6). However, not all studies confirmed the associations. In the Framingham Study in adults, urate was not associated with adverse cardiovascular events after adjustment for cardiovascular disease risk factors (7). Similarly, NHANES III study, again performed in adults, showed that serum urate was not associated with cardiovascular or chronic heart disease related mortality (8).

**Figure 1:**
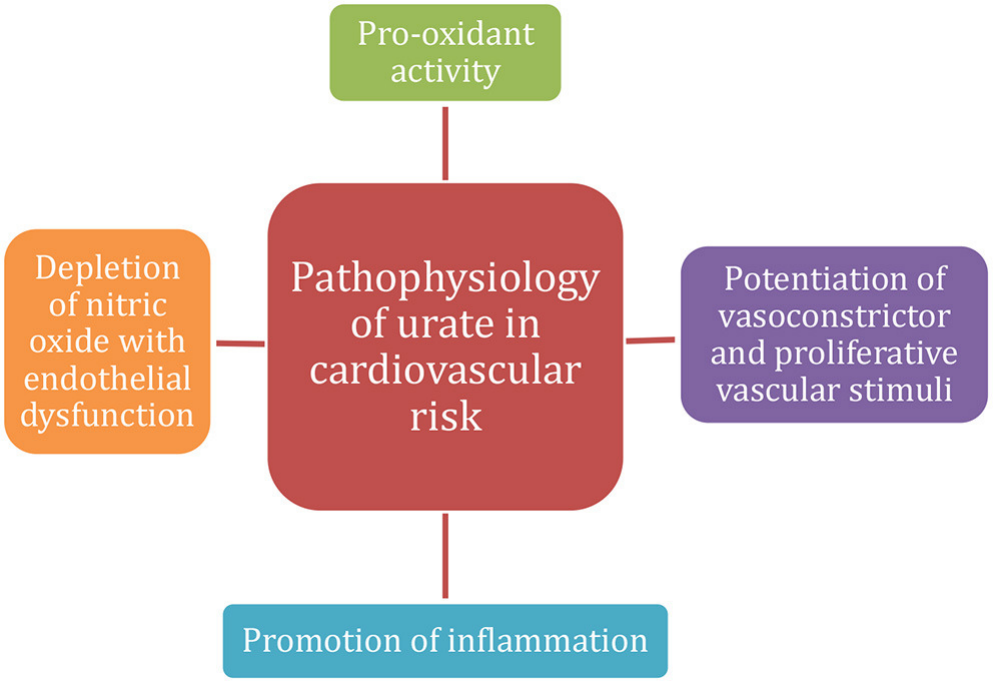
Proposed mechanisms of urate pathophysiology in cardiovascular risk.

The proposed explanations for urate and cardiovascular risk factors associations are presented in Figure 1. Generally, accepted mechanisms of involvement include pro-oxidant activity, depletion of nitric oxide with endothelial dysfunction, promotion of inflammation and potentiation of vasoconstrictor and proliferative vascular stimuli (9).

In the pediatric population, hyperuricemia was also common in children with chronic kidney disease and was associated with renal dysfunction, hypertension, obesity and albuminuria (10). Consistent with animal model data and evidence in the adult population, the role of urate in hypertension development has been strongly supported, but only in some cases of early-onset hypertension (11–13). Hyperuremic hypertension, supported by animal models, proposed two-phase mechanism for its development including vasoconstriction by activation of the renin-angiotensin system via angiotensin II activation and reduction of circulating nitric oxide, which can be reversed by lowering uric acid (11, 14). Hyperuricemia in children has also been strongly associated with overweight and obesity (15). In addition, an association between fructose consumption, increased urate production and increased cardiovascular risk has been established at pediatric age (16).

In adults, many studies of urate evaluate its effects on cardiovascular morbidity and mortality. In young persons, they are less often expected, however, some signs of organ damage, such as left ventricular hypertrophy (17), can already be seen in the pediatric population and present risk for future cardiovascular events at a young age. To evaluate prognostic value of urate level in the young, subclinical atherosclerosis evaluation could provide additional information. Thus, associations of urate with vascular markers of subclinical atherosclerosis (carotid artery intima-media thickness, carotid plaque, carotid distensibility and brachial flow-mediated dilatation) were examined in young adults between 30 and 45 years of age. Associations of urate and cardiovascular risk markers, especially body mass index (BMI) were found, however, there was no evidence of urate having an independent role in the pathophysiology of early atherosclerosis (18).

The role of urate in cardiovascular risk development in young individuals remains elusive, at least in providing its prognostic value. The aim of our study was to evaluate the associations between serum urate and pulse wave velocity (PWV), another measure of subclinical atherosclerosis evaluation (19, 20), in adolescents and young adults with hypertension.

## Materials and Methods

### Subjects

A total of 269 adolescents and young adults were included in the study. Subjects were diagnosed with hypertension and additionally evaluated for other cardiovascular risk factors. The diagnosis was made according to diagnostic guidelines and age dependent cut off values (21). The data for this study were analyzed retrospectively. We examined our PWV measurements and included all adolescents and young adults with hypertension for whom all necessary measurements and serum urate were available.

The study of PWV measurement was approved by the Institutional Ethics Committee as was their retrospective analysis with inclusion of other acquired data. Laboratory investigations were performed as part of routine follow-up. Subjects were additionally labeled as obese (with BMI above 95^th^ percentile according to International Obesity Task Force (IOTF) (22)) or non-obese; 155 subjects were normal- or overweight and 114 were obese. Moreover, subjects were divided to those with elevated (131 subjects) or those with normal urate (138 subjects) according to age and gender specific reference values (2), since urate also changes with age (2).

### Data Collection

The following data were collected for each participant: age, height, weight, BMI, systolic blood pressure (SP), diastolic blood pressure (DP), PWV and serum urate.

PWV was measured using arterial applanation tonometry (SphygmoCor, SCOR-Vx, Australia). All measurements were performed according to the manufacturer's instructions by three trained investigators with good intra-observer reliability. Before the measurement, each subject's weight and height, blood pressure and the arterial path length between the two recording sites (e.g., radial and carotid artery) were measured. Next, a pressure tonometer was used to transcutaneously record the pressure pulse waveform at the recording site. An ECG signal provided a timing reference for the software to be able to calculate PWV (23, 24).

SPSS Statistics (IBM, version 22) was used for statistical analyses. The statistical tests used included multiple regression analysis and independent samples *T* test. Additionally, for the discussion, correlations with Pearson correlation coefficient between urate and variables were performed in only normal-weight participants, data are not shown. A value of *p* < 0.05 was considered statistically significant.

## Results

The characteristics of the studied population are presented in Table 1.

**Table 1:** The characteristics of the studied population.

**Variable**	**Mean (min, max) ± SD**
Age [years]	15.5 (11, 22) ± 2.4
Height [cm]	171 (131, 197) ± 11.3
Weight [kg]	79 (31, 165) ± 22
BMI [kg/m^2^]	26.7 (14.5, 53.9) ± 6.4
Systolic pressure [mmHg]	135 (93, 182) ± 13
Diastolic pressure [mmHg]	78 (56, 112) ± 10.6
PWV [m/s]	6.49 (4.0, 10.4) ± 1.11
Serum urate [μmol/L]	313 (139, 506) ± 75

*BMI, body mass index; PWV, pulse wave velocity*.

Next, we compared variables according to sex, between obese and non-obese and between normal and elevated urate level with independent samples *T* test. All the results are presented in Table 2. Variables are presented as mean ± SD.

**Table 2:** Comparison of included parameters between obese and non-obese participants, and participants with normal or elevated urate.

	**Boys (*N* = 184)**	**Girls (*N* = 85)**	**Non-obese (*N* = 155)**	**Obese (*N* = 114)**	**Normal urate (*N* = 131)**	**Elevated urate (*N* = 138)**
Age	15.7 ± 2.2	15.1 ± 2.7	16 ± 2.4	14.9 ± 2.3	15.8 ± 2.5	15.3 ± 2.3
	*p* = 0.078		*p* <0.001		*p* = 0.104	
Height	175 ± 10.4	163 ± 7.7	172 ± 11	170 ± 11	170 ± 12	172 ± 10
	*p* <0.001		*p* = 0.213		*p* = 0.150	
Weight	83 ± 21.7	71 ± 19.9	67.3 ± 13.2	94.9 ± 21	72 ± 19	86 ± 22
	*p* <0.001		*p* <0.001		*p* <0.001	
BMI	26.7 ± 6	26.7 ± 7	22.5 ± 2.7	32.4 ± 5.3	24.5 ± 5.5	28.8 ± 6.4
	*p* = 0.952		*p* <0.001		*p* <0.001	
SP	137 ± 13	130 ± 12	134 ± 14	135 ± 12	136 ± 15	135 ± 12
	*p* <0.001		*p* = 0.801		*p* = 0.504	
DP	78 ± 10	79 ± 11	79 ± 11	76 ± 10	80 ± 11	77 ± 9
	*p* = 0.242		*p* = 0.034		*p* = 0.009	
PWV	6.4 ± 1.1	6.6 ± 1	6.6 ± 1.1	6.3 ± 1.1	6.6 ± 1.1	6.4 ± 1.1
	*p* = 0.281		*p* = 0.061		*p* = 0.162	
Serum urate	335 ± 67	265 ± 68	290 ± 69	343 ± 71	257 ± 49	366 ± 53
	*p* <0.001		*p* <0.001		*p* <0.001	

*BMI, body mass index; SP, systolic pressure; DP, diastolic pressure; PWV, pulse wave velocity*.

Regarding obesity, in addition to anthropometrical parameters, significant differences were found for diastolic blood pressure and urate. Regarding hyperuricemia, significant differences were found for BMI and diastolic blood pressure.

Next, correlation tests, presented in Table 3, for urate and other variables were conducted between normal-uremic and hyper-uremic group. In both, urate correlated with growth parameters and systolic blood pressure, although in one group urate was significantly higher. However, pulse wave velocity did not correlate to urate in neither of the two groups.

**Table 3:** Correlations between urate and other studied variables in cohort of normal-uremic and hyper-uremic participants.

**Correlation coefficients [r] and probability [*p*]**	**Age**	**Height**	**Weight**	**BMI**	**SP**	**DP**	**PWV**
Normal-uremic group	r = 0.275 *p* = 0.001	r = 0.499 *p* <0.001	r = 0.542 *p* <0.001	r = 0.320 *p* <0.001	r = 0.206 *p* = 0.018	r = −0.158 *p* = 0.072	r = −0.028 *p* = 0.751
Hyper-uremic group	r = 0.073 *p* = 0.394	r = 0.364 *p* <0.001	r = 0.350 *p* <0.001	r = 0.203 *p* = 0.017	r = 0.179 *p* = 0.036	r = −0.038 *p* = 0.656	r = −0.108 *p* = 0.206

*BMI, body mass index; SP, systolic pressure; DP, diastolic pressure; PWV, pulse wave velocity; r, Pearson correlation coefficient*.

Multiple regression analysis with urate as a dependent variable, presented in Table 4, included gender, age, height, weight, BMI, systolic pressure, diastolic pressure and PWV. Adjusted R^2^ for the model was 0.382 with a standard error of the estimate of 58.65. Gender and diastolic pressure were shown to be statistically significant in the model.

**Table 4:** Multiple regression analysis with urate as dependent variable.

**Variables**	**β**	***p*-value**	
Gender	−0.33	<0.001	
Age	−0.071	0.224	
Height	0.11	0.597	
Weight	0.177	0.682	(F value = 21.69, *p* <0.001)
BMI	0.242	0.517	
SP	0.005	0.932	
DP	−0.121	0.029	
PWV	−0.065	0.192	

*BMI, body mass index; SP, systolic pressure; DP, diastolic pressure; PWV, pulse wave velocity*.

## Discussion

In our study, we focused on possible associations between urate and cardiovascular risk, assessed by PWV. We recruited adolescents and young adults with hypertension and found important associations between urate and gender, obesity and diastolic blood pressure, but no associations between urate and PWV.

### Urate and Gender

In adults, important sex difference between urate levels was observed already more than 40 years ago (25, 26), as in our study. The most obvious explanation is the hormonal difference between the men and women that becomes apparent after puberty starts, which is consistent with our findings. The same was the idea more than 40 years ago, and therefore the role of estradiol-17β was investigated, however, not confirmed (26). The architecture of the female kidney is likely distinct from that of the male kidney since being a woman seems to be protective from renal and cardiovascular disease with better outcomes (27). A recent study determined that females with chronic kidney disease have a slower decline in glomerular filtration rate and a lower risk of death compared to age-matched men with similar mild-to-moderate chronic kidney disease (28). Studies have shown that estrogen may play a role in the regulation of expression or activity of urate transporters, which is supported by the fact that females have different renal urate transporter expression, localization or activity. Estrogen could mediate either direct transcriptional regulation of the transporter genes, or activate transporter specific transcription factors (27). Consequently, the levels of estrogen were inversely correlated to the levels of urate in post-menopausal women, in transgender males (female to male) and even in urate level changes during ovulation, when estrogen is lowest. The urate was also lowered when estrogen was administered after menopause (27, 29–31).

Studies of the adult population cannot simply be transferred to the pediatric population. Pediatric studies in this field are lacking, however, an interesting study demonstrated that the increase in urate at the time of puberty might be also related to muscle mass. Girls with higher muscle mass, evaluated by multi-frequency bioelectrical impedance, had higher urate levels as did boys, where the effect of muscle mass was even higher. Therefore, the difference in urate between boys and girls might be partially a direct influence of muscle mass, affected by hormonal changes, specifically with testosterone in boys (32). Additionally, association between urate and testosterone was found among obese children, indicating an impact of androgens in the regulation of serum urate in obesity (33).

### Urate and Obesity

Hyperuricemia has been frequently associated with obesity, renal disease, hyperlipidaemia, atherosclerosis and hypertension (34). In our study, significantly higher urate levels were found in patients with obesity, confirming the previously published studies. The increase in serum urate has often been considered to be secondary, however, some recent findings suggested that it may have a contributory role, because elevated serum urate levels preceded obesity (35).

Similarly, obesity and urate levels were associated in children, both in boys and girls, where significant differences between urate levels were observed during puberty. Obese children had elevated urate levels already at the beginning of the puberty indicating that obesity may affect urate's metabolism with either renal retention of urate, different modulation of urate at the tubular level and at least in part due to the urate overproduction in obese individuals (36, 37). Obesity is commonly associated with increased risk of cardiovascular disease, however, approximately a third of obese individuals do not develop cardiovascular disease (they are so called “metabolically healthy obese”). Recently, serum urate was found to be a good predictor of “metabolically unhealthy obesity” with associated features of metabolic syndrome, suggesting increased cardiovascular risk in adolescents and adults and might be useful as a prognostic marker (3).

### Urate and Blood Pressure

Other cardiovascular risk factors, such as blood pressure, lipid profile and insulin levels were associated with higher urate levels. Multiple regression analyses in the above-mentioned study showed BMI, triglycerides, cholesterol LDL and insulin to be significant predictors of increased urate (38), however, not blood pressure, which is frequently associated with hyperuricemia (12). In our cohort, all participants have been diagnosed with hypertension and many were taking antihypertensive medications at the time of the measurement and laboratory work-up. Therefore, their blood pressure was already influenced by external factors making them difficult to evaluate. We would expect higher values of both SP and DP among hyperuricemia patients, as in other studies (13), where urate-lowering therapy even resulted in significant reduction in blood pressure (14). Anyway, the association with diastolic blood pressure was confirmed with multiple regression analysis. According to studies, hyperuricemia in hypertensive adolescents and young adults might not be associated with only obesity-associated hypertension, but could be in some cases independent risk factor for hypertension development in normal-weight individuals (12).

### Urate and Cardiovascular Risk

Hyperuricemia in cardiovascular risk is commonly asymptomatic in contrast to acute hyperuricemia in gout. The question remains if hyperuricemia in this context should be treated or not. Several associations between urate and cardiovascular diseases have already been mentioned. In adults, urate was studied also as a prognostic tool where it was found a good marker of mortality in patients with stable coronary artery disease treated with percutaneous coronary interventions, patients with chronic obstructive pulmonary disease and terminally ill cancer patients (39).

In adolescents and young adults, the prognostic value of urate and the need to treat hyperuricemia is less clear. So far, we can associate its role in some cases of essential hypertension development and in obesity by delineating “metabolically unhealthy” adolescents and young adults with increased cardiovascular risk. Little is known how elevated urate affects atherosclerosis itself, the common denominator of cardiovascular diseases.

In our study, we aimed to assess the associations between urate and PWV, a marker of arterial stiffness, which was associated with most cardiovascular risk factors and established atherosclerosis in adults (40), as well as in children with hypertension, obesity, impaired lipid profile and glucose metabolism (19, 41–44). Elastic properties of arteries change over time due to the normal aging and due to the pathological processes, such as presence of cardiovascular risk factors that can accelerate atherosclerosis. Higher arterial stiffness is associated with higher cardiovascular mortality, chronic heart disease, and stroke in adults (45, 46). Some associations between urate and PWV in adults have been already studied, however, the results are not consistent. One study found correlation between urate levels with greater increase in PWV in men but not women, which was lost when men with hyperuricemia were not included (47). In a meta-analysis, a significant association between urate and carotid-femoral-PWV was found in the general population in both males and females while it was only significant for females regarding brachial-ankle-PWV. Additionally, in a few available studies no significant relationship was found between urate and both carotid-femoral- and brachial-ankle-PWV in hypertensive patients (48). On the contrary, a recent study demonstrated an association between urinary, not serum urate, and arterial stiffness in adult men (49).

Urate was associated with increased arterial stiffness in stroke survivors in adult population, independently of other risk factors (50). Decreasing urate with allopurinol did not seem to be effective in reducing stiffening, but febuxostat showed an effect in decreasing PWV (51). A therapeutic intervention trial of lowering of serum urate is required to see whether this will affect positively on mortality and morbidity in patients with high cardiovascular risk. In adults, xanthine oxidase blocking treatment can be considered to reduce the risk of all-cause mortality in patients with higher cardiovascular risk and hyperuricemia along with recommended lifestyle modifications (39).

The studies investigating associations between urate and arterial stiffness in the pediatric population are scarce, let alone the evaluation of its prognostic role in future cardiovascular events. In our study, we did not confirm any associations between urate and arterial stiffness in a cohort of hypertensive adolescents and young adults, who are at increased risk for subclinical atherosclerosis. Similarly, there were no associations between urate and arterial stiffness in a cohort of adolescents with type 1 diabetes (52).

However, in our study it might be challenging to separate the effect of urate on pulse wave velocity from those due to hypertension, obesity and age. Additionally, blood pressure modifications during measurement, such as antihypertensive modifications and stress, might influence PWV making our study deficient with larger sample of participants required to eliminate shortcomings.

To our knowledge, no other similar studies were conducted and further research is needed to evaluate the prognostic role of urate in adolescents and young adults at cardiovascular risk, especially in hypertension or obesity, where the role of urate seems to be more important.

## Conclusion

In our study, we evaluated the associations between serum urate and pulse wave velocity, a measure of arterial stiffness and cardiovascular risk, in a cohort of adolescents and young adults with hypertension. Associations between urate, gender and obesity were confirmed, however, no significant association between pulse wave velocity and urate was detected.

Our results indicate that in adolescents and young adults the effect of urate on the cardiovascular system, recognized in the adult population, is not as pronounced, but opens a window of opportunity for lifestyle modifications before development of cardiovascular complications and the need for pharmacological therapy in adult life.

## Data Availability Statement

The raw data supporting the conclusions of this article will be made available by the authors, without undue reservation.

## Ethics Statement

The studies involving human participants were reviewed and approved by Institutional Ethics Committee of University Medical Centre Maribor (UKC-MB-KME-25/16 and UKC-MB-KME-58/21). Written informed consent to participate in this study was provided by the participants' legal guardian/next of kin.

## Author Contributions

MM and NMV: conceptualization. MM: methodology, formal analysis, investigation, writing—original draft preparation, and visualization. MM, SGJ, MF, and NMV: patient management and data acquisition. SGJ, MF, and MM: pulse wave velocity measurements. NMV: resources, supervision, project administration, and funding acquisition. SGJ, MF, and NMV: writing—review and editing. All authors have read and agreed to the published version of the manuscript.

## Conflict of Interest

The authors declare that the research was conducted in the absence of any commercial or financial relationships that could be construed as a potential conflict of interest.

## Publisher's Note

All claims expressed in this article are solely those of the authors and do not necessarily represent those of their affiliated organizations, or those of the publisher, the editors and the reviewers. Any product that may be evaluated in this article, or claim that may be made by its manufacturer, is not guaranteed or endorsed by the publisher.
